# Superusers in Social Networks for Smoking Cessation: Analysis of Demographic Characteristics and Posting Behavior From the Canadian Cancer Society's Smokers' Helpline Online and StopSmokingCenter.net

**DOI:** 10.2196/jmir.1854

**Published:** 2012-06-26

**Authors:** Trevor van Mierlo, Sabrina Voci, Sharon Lee, Rachel Fournier, Peter Selby

**Affiliations:** ^1^Evolution Health Systems Inc.San Francisco, CAUnited States; ^2^Nicotine Dependence ClinicCentre for Addiction and Mental HealthToronto, ONCanada; ^3^Canadian Cancer Society, Ontario DivisionToronto, ONCanada; ^4^Dalla Lana School of Public HealthUniversity of TorontoToronto, ONCanada

**Keywords:** Social networks, moderated support, eHealth, community, smoking cessation, Web-assisted tobacco anterventions, WATI

## Abstract

**Background:**

Online social networks are popular components of behavior-change websites. Research has identified the participation of certain network members who assume leadership roles by providing support, advice, and direction to other members. In the literature, these individuals have been variously defined as *key players*, *posters*, *active users*, or *caretakers*. Despite their identification, very little research has been conducted on the contributions or demographic characteristics of this population. For this study, we collectively categorized key players, posters, active users, and caretakers as superusers.

**Objectives:**

To analyze data from two large but distinct Web-assisted tobacco interventions (WATI) to help gain insight into superuser demographic characteristics and how they use social networks.

**Methods:**

We extracted cross-sectional data sets containing posting behaviors and demographic characteristics from a free, publicly funded program (the Canadian Cancer Society’s Smokers’ Helpline Online: SHO), and a free, privately run program (StopSmokingCenter.net: SSC).

**Results:**

Within the reporting period (SHO: June 26, 2008 to October 12, 2010; SSC: May 17, 2007 to October 12, 2010), 21,128 individuals registered for the SHO and 11,418 registered for the SSC. Within the same period, 1670 (7.90%) registrants made at least one post in the SHO social network, and 1627 (14.25%) registrants made at least one post in the SSC social network. SHO and SSC superusers accounted for 0.4% (n = 95) and 1.1% (n = 124) of all registrants, and 5.7% (95/1670) and 7.62% (124/1627) of all social network participants, and contributed to 34.78% (29,422/84,599) and 46.22% (61,820/133,753) of social network content, respectively. Despite vast differences in promotion and group management rules, and contrary to the beliefs of group moderators, there were no statistically significant differences in demographic characteristics between the two superuser groups.

**Conclusions:**

To our knowledge, this is the first study that compared demographic characteristics and posting behavior from two separate eHealth social networks. Despite vast differences in promotional efforts and management styles, both WATI attracted superusers with similar characteristics. As superusers drive network traffic, organizations promoting or supporting WATI should dedicate resources to encourage superuser participation. Further research regarding member dynamics and optimization of social networks for health care purposes is required.

## Introduction

Tobacco-related illnesses are the leading cause of death in North America, yet 46 million (21%) Americans [1] and 6 million (18%) Canadians [2] continue to smoke. Despite decades of research and prevention, the World Health Organization predicts that tobacco-related illnesses will cause 10 million deaths per year by 2030 [3].

In traditional treatment for tobacco dependence, extensive evidence proves the effectiveness of behavioral interventions such as brief or intensive advice, individual or group counseling, tailored self-help, and telephone quitlines [4,5]. However, despite their proven effectiveness, evidence also shows that these interventions are vastly underused [6]. What is required are effective interventions that have high reach and are easily accessible, implemented, and maintained [7].

As of June 30, 2010, it is estimated that 29% of the world’s population has access to the Internet [8]. Research has shown that increasing numbers of individuals are accessing the medium for general health information and to seek assistance with specific addiction and mental health concerns [9,10]. Based on this uptake, a large number of randomized controlled trials and observational studies have investigated how Internet-based interventions can successfully help individuals with problem drinking [11-13], mood and anxiety disorders [14-17], and other conditions [18,19].

### Online Social Support for Tobacco Cessation

Following this trend, Web-assisted tobacco interventions (WATI) are proving to be efficacious [20-22]. A popular component of WATI is a social network, comprising online communities of people with a common interest who use a website to communicate with each other, also commonly known as *support groups*, *user forums*, or *discussion boards*. Although there is some evidence that social networks can potentially enhance effectiveness and adherence to eHealth interventions [23,24], very little research has been conducted on how social networks function or who accesses them. There are no best practices for their implementation or maintenance.

In a 2010 study, Cobb et al [25] identified the consistent contributions of specific members over time in QuitNet, a large online community for smoking cessation. In 2010, Selby et al [24] found that 25% of first posts made within StopSmokingCenter.net (SSC) were from recent quitters who were struggling with their quit attempts. Their study also found that 35.0% of first replies were from members who had quit within the past month, 49.0% were from members who had quit for more than 1 month but less than 1 year, and 6.6% of first responses to new messages were from experienced members who had quit for more than a year [24]. In a 2007 analysis of AlcoholHelpCenter.net, an online social network for problem drinkers, Cunningham et al [26] found that discussions clustered around nodes of one or more active users. Most recently in 2011, Jones et al [27] identified members in a self-harm discussion forum (SharpTalk) who logged on for much greater times than others and mainly posted in response to other participants.

Social network members who assume leadership roles by providing direct support, advice, and direction are defined by Cobb et al as *key players*, by Selby et al as *posters*, by Cunningham et al as *active users*, and by Jones et al as *caretakers*. For consistency in this study we collectively define key players, posters, active users, or caretakers as *superusers*. The purpose of this observational study was to analyze data from two large but distinct WATI to help gain insight into superuser demographic characteristics and how they use social networks.

### The Labyrinth of eHealth Applications Online: Promotion and Adherence

While the potential to help individuals through legitimate and validated eHealth interventions is exciting, the explosive growth of Internet access parallels the ever-increasing number of websites and Internet protocols (or IPs or URLs, commonly referred to as *domain names *or *IP addresses*). The overwhelming number of websites available can be especially confusing for general Internet users when distinguishing between legitimate and nonlegitimate eHealth resources [28]. Because the vast majority of search engines and directories generate revenue by promoting website URLs during general searches, and advertisers can maximize their budgets by using algorithms that deliver appealing campaigns to specific demographics and psychographics, it may become increasingly difficult and expensive for legitimate eHealth resources to attract new users.

One method for legitimate eHealth interventions to attract users is through directories such as Beacon [29], an Australia-based organization that publicizes only eHealth IPs that have undergone randomized controlled trials and pass an independent review from a panel of experts. The challenge for important resources such as Beacon is that they require consistent international funding, promotion, and cross-collaborative support to remain effective. Users may also seek new, innovative technologies that may be missed by such directories, since publishing outcome data from randomized controlled trials lags behind adoption of emerging tools.

An alternative grassroots method that can attract general users is through social networking and the retention of superusers. As they expand the size of a network and facilitate discussion, superusers are valuable assets for eHealth social networks to recruit and retain.

For organizations implementing and managing online social networks, identifying and retaining superusers could contribute to the natural development and growth of website traffic and promote adherence. The challenge is determining how to identify superusers, how to attract them, and how to promote their retention. The first step in this process is to begin to understand who superusers are.

## Methods

### Setting and Program Description

**Figure 1:**
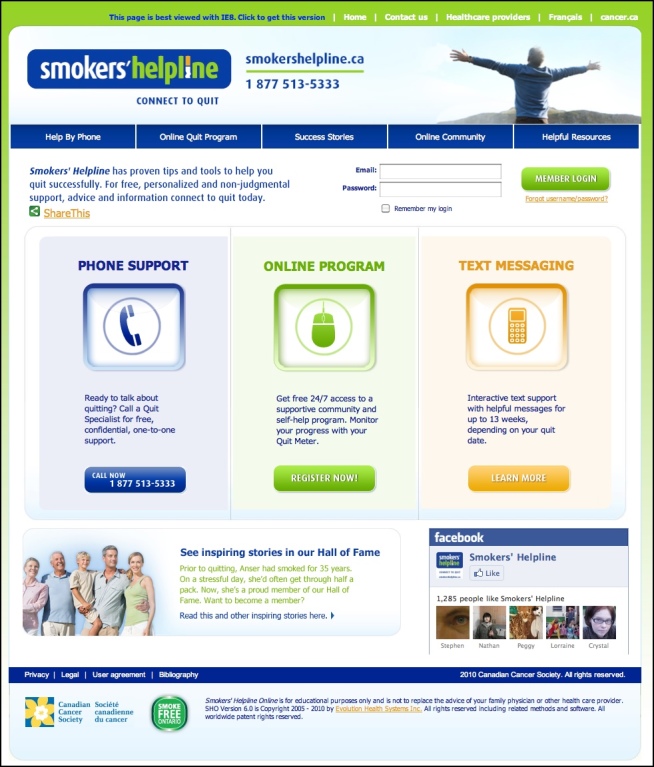
SmokersHelpline.ca version 6.0 home page.

**Figure 2:**
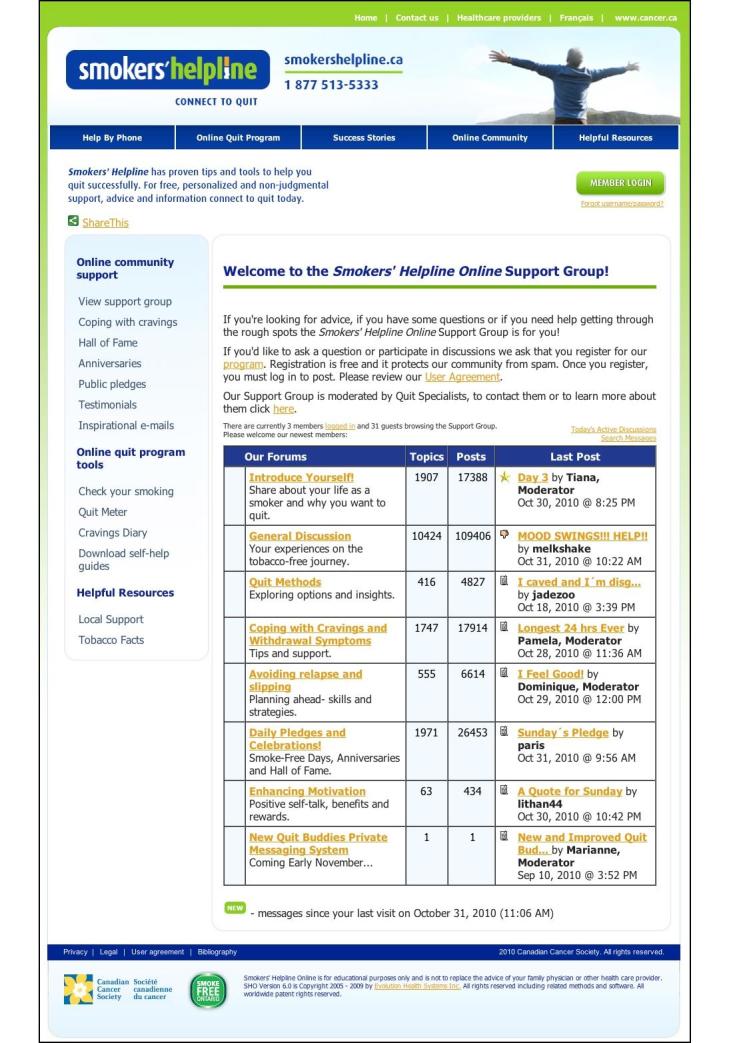
SmokersHelpline.ca version 6.0 support group home page.

**Figure 3:**
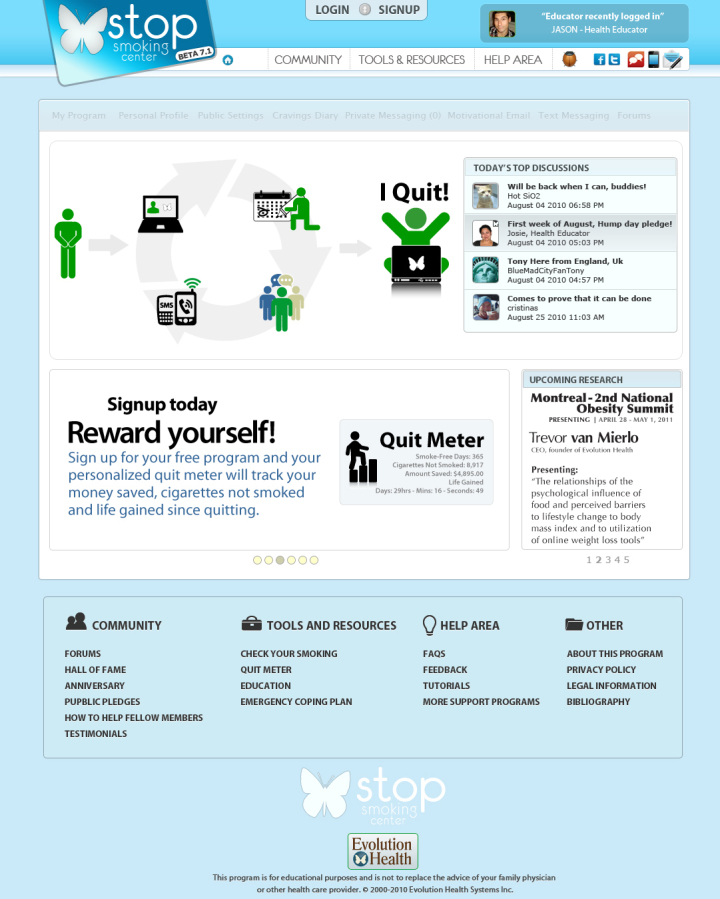
StopSmokingCenter.net version 7.1 home page.

**Figure 4:**
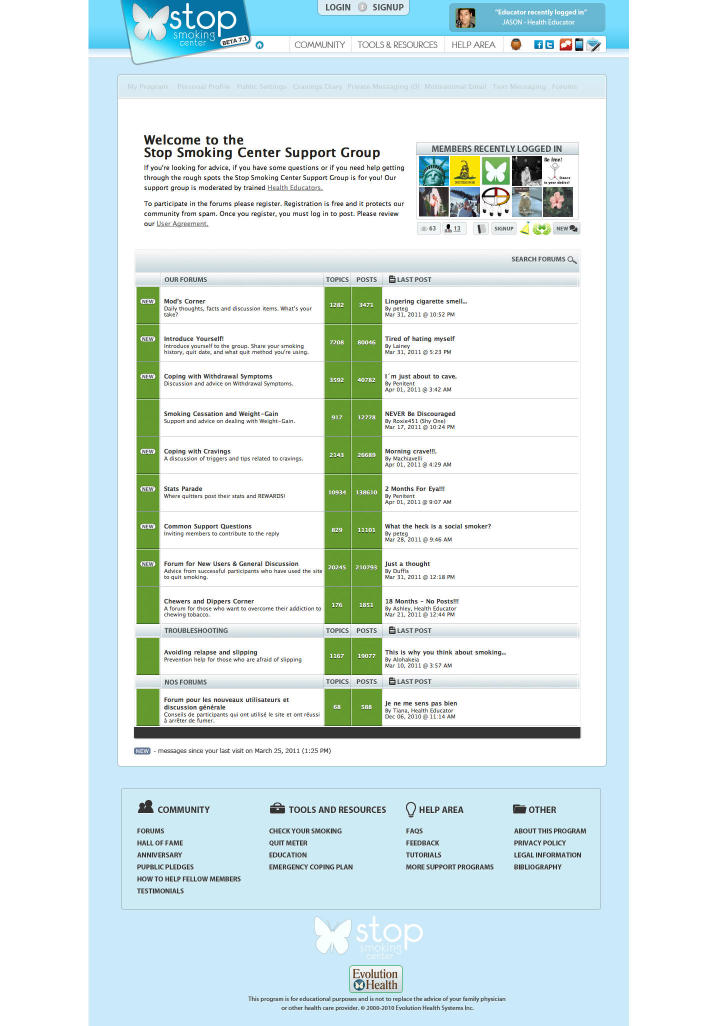
StopSmokingCenter.net version 7.1 support group home page.

As an initial step designed to investigate superuser characteristics, this observational study analyzed data from two large social networks for smoking cessation: the Canadian Cancer Society’s Smokers’ Helpline Online (SHO) (http://smokershelpline.ca; Figure 1, Figure 2) [30], and the SSC (http://www.stopsmokingcenter.net; Figure 3, Figure 4) [31]. Both programs are available free of charge and are anonymous. We chose the SHO and the SSC social networks due to their large size, active participation, and considerable operational differences.

### The Canadian Cancer Society’s Smokers’ Helpline Online

As a publicly funded program with an internationally recognized and respected brand, SHO undertakes substantial promotional campaigns extending to Internet, television, radio, and print advertising, attracting many smokers. The SHO social network is also used to promote offline activities, such as The Ontario Driven to Quit Challenge. Also, SHO program health educators are required to rigorously monitor content posted by members. An unlimited number of individuals may register for the SHO program. The SHO is a licensed version of the SSC that is modified and updated by the Canadian Cancer Society.

### StopSmokingCenter.net

Contrarily, the SSC is not a recognized brand, has never been advertised or promoted, and is maintained as a social enterprise. The organization that operates the SSC (Evolution Health System Inc, San Francisco, CA, USA) does not actively promote the program, nor does it optimize search engine rankings or actively seek links from other websites. Therefore, members using the SSC are a self-seeking, naturalistic Internet population. Although users must endorse a user agreement to participate in the SSC social network, unlike in the SHO, health educators control very little content posted by members.

### SHO and SSC Social Network History, Functionality, and Health Educator Roles

To actively participate in each social network, registration is required. However, actively participating in the social networks is voluntary; in the study period only 7.90% (n = 1670) of SHO registrants and 14.25% (n = 1627) of SSC registrants made at least one post in the social network (see Table 1). Program registrants and lurkers do not have to register to browse member conversations within each social network.

**Table 1:** Total program registrations and social network participation in Smokers’ Helpline Online (SHO) and StopSmokingCenter.net (SSC) Web-assisted tobacco interventions.

Characteristic	SHO	SSC
Study period	June 26, 2008–October 12, 2010	May 17, 2007–October 12, 2010
Duration (days)	839	1245
Number of registrants	21,128	11,418
Members with at least one post in the social network, n (%)	1670 (7.90%)	1627 (14.25%)

Every post made in each support group is published immediately. However, to ensure compliance with program rules and regulations, trained health educators review and approve each post through WebTriage (Evolution Health System Inc, San Francisco, CA, USA), a software application designed to facilitate the rapid approval, editing, or deletion of each post.

SHO health educators are paid employees who also manage the Canadian Cancer Society’s telephone quitline. SSC health educators are paid employees of Evolution Health Systems Inc. In addition to training in the use of WebTriage, all health educators receive training in cognitive behavioral therapy, stages of change, motivational interviewing, life coaching, data protection, and user privacy. During high-volume seasons, SSC health educators assist SHO health educators with moderating the SHO social network (WebTriage).

### Informal Qualitative In-Person Interviews With Social Network Health Educators

For over 5 years, SHO and SSC social network managers have met together on a bimonthly basis to discuss social network management policies, specific incidences within each support group, moderating strategies designed to increase social network traffic, and the management of superuser populations.

On a quarterly basis, SHO and SSC social network managers meet to review cumulative statistical reports, which include the number of program registrants, number of social network posts, traffic statistics (page views, visits, and unique visitors), and basic demographic data collected at registration.

At the onset of this study, we conducted informal qualitative, in-person interviews with health educators and their management teams. In these interviews, we asked interviewees to reflect on bimonthly and quarterly meetings, each program’s promotional and operational differences, and each network’s tone and social environment. In these interviews all health educators felt that each program attracted superusers with heterogeneous characteristics, and that in each social network posting behavior and demographics would be correlated.

However, health educator and social network managers indicated that, based on their years of experience, they could not generalize superusers into a single category, and that three distinct types of superusers existed: (1) superusers who cumulatively authored the greatest number of posts (*posters*), (2) superusers who mainly initiated threads (*thread starters*), and (3) superusers who only communicated with certain members (*clique members*), and that management styles differed for each of these superuser subgroups.

### Ethical Considerations

All study participants consented to the use of their anonymous data for research purposes. Data collection procedures adhered to international privacy guidelines [32-34] and were in accordance with the Helsinki Declaration of 1975, as revised in 2008 [35]. As the study was based on unidentifiable archival data, the study was deemed to be exempt from further review.

### Participants

Registration to both SHO and SSC is anonymous and free of any commitment or fees. From June 26, 2008 to October 12, 2010 (839 calendar days), 1670 members posted at least once in the SHO social network, and there were 84,599 posts made in 7916 threads. From May 17, 2007 to October 12, 2010 (1245 calendar days), 1627 members posted at least once in the SSC social network, and there were 133,753 posts made in 10,967 threads (see Table 2). During the study periods both programs used identical question formats and response options in the analysis of demographic characteristics.

**Table 2:** Total social network activity in Smokers’ Helpline Online (SHO) and StopSmokingCenter.net (SSC) Web-assisted tobacco interventions.

Characteristic	SHO	SSC
Study period	June 26, 2008–October 12, 2010	May 17, 2007–October 12, 2010
Duration (days)	839	1245
Number of members with at least one post in the social network	1670	1627
Number of posts in the social network	84,599	133,753
Number of message threads	7916	10,967
Number of posts in shortest thread	1	1
Number of posts in longest thread	73	87

### Data Collection

Users completed a baseline questionnaire upon program registration. Questions were based on the North American Quitline Consortium Minimal Data Set [36]. All data were self-reported. On registration, each user is assigned an anonymous user identification, which becomes his or her primary key. We extracted cross-sectional data sets containing posting behaviors and demographic characteristics from each program’s customized Structured Query Language database. Health educators’ posts were excluded from analysis. Users’ primary keys linked their posting behavior with their demographic data.

### Posting Behavior and Social Network Participation

**Figure 5:**
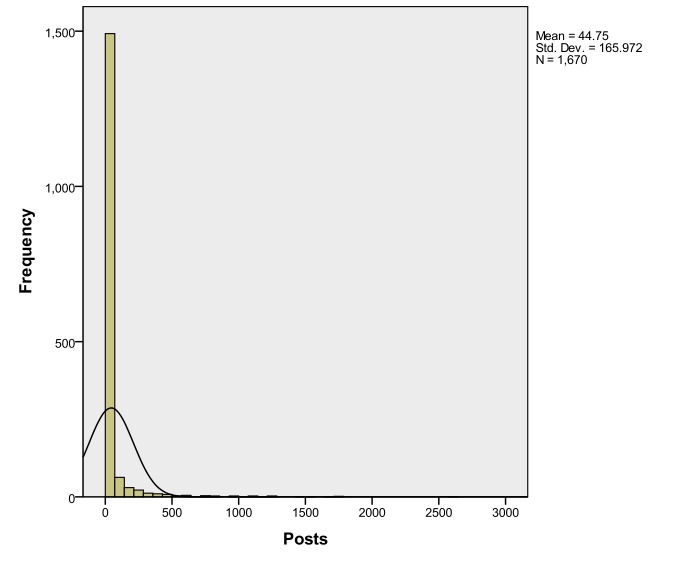
Posting trends in smokershelpline.ca.

**Figure 6:**
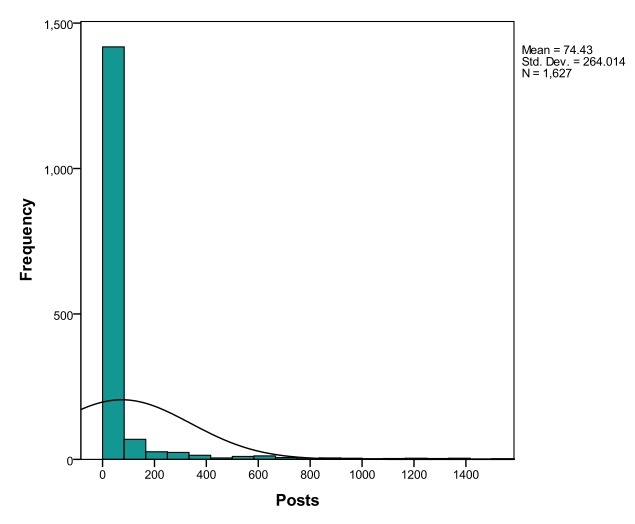
Posting trends in StopSmokingCenter.net.

In accordance with observations by Cobb et al [25], Selby et al [24], Cunningham et al [26], and Jones et al [27], analysis of posting behavior in both social networks revealed right-skewed distributions, meaning that, cumulatively, most users posted infrequently, and that superusers were responsible for the majority of posts (see Figure 5 and Figure 6).

### Superuser Definition

In each social network, we combined the top 100 posters (ranked according to their total number of posts), thread starters (ranked according to the total number of threads they started), and clique members (ranked according to the number of threads they participated in) in a single database (n = 300). Duplicate entries were removed, leaving a sample of 219 unique superusers. We conducted a power analysis [37] and estimated a total sample size of 95 SHO and 124 SSC superusers to have a power >80% to test the hypothesis at the *P *< .05 level of significance.

### Data Analysis

At registration, SHO and SSC used identical formats and response options for the following demographic variables: gender, age, cigarettes per day, past quit attempts, cohabitant smokers, years smoked, and past nicotine replacement therapy usage. Both programs collected other demographic data, but question formats or response options differed significantly, and thus we excluded those data from analysis.

Descriptive statistics pertaining to general posting behavior and demographic characteristics of both superuser groups are presented first. Next, we conducted sets of univariate logistic regressions to detect differences in demographic characteristics between SHO and SSC superusers. Finally, Pearson correlations were computed to detect relationships between behavior and demographic characteristics in each superuser subgroup. All analyses were performed using SPSS for Windows version 18.0 (IBM Corporation, Somers, NY, USA). The significance level was set at *P *< .05.

## Results

### General Posting Behavior and Demographic Characteristics

The 95 SHO and 124 SSC superusers accounted for 0.4% and 1.1% of total program registrants and 5.7% and 7.6% of all active social network members. They were responsible for 34.78% (n = 29,422) and 46.22% (n = 62,820) of all social network posts (see Table 3).

Analysis of demographic characteristics collected at registration (means) and their standard deviations revealed, from the perspective of marketing and moderating techniques, slightly distinct populations (see Table 4).

**Table 3:** Superuser social network activity in Smokers’ Helpline Online (SHO) and StopSmokingCenter.net (SSC) Web-assisted tobacco interventions.

Characteristic	SHO	SSC
Total number of superusers	95	124
Percentage of total registrants	0.4%	1.1%
Percentage of active social network members	5.7%	7.6%
Total number of posts in social network	84,599	133,753
Posts in social network by superusers, n (%)	29,422 (34.78%)	61,820 (46.22%)

**Table 4:** Superuser demographic characteristics in Smokers’ Helpline Online (SHO) versus StopSmokingCenter.net (SSC) Web-assisted tobacco interventions.

Characteristic	SHO	SSC
Gender (female), n (%)	79 (83%)	91 (73%)
Age (years), mean (SD)	46.0 (11.5)	48.6 (9.5)
Cigarettes smoked per day, mean (SD)	20.3 (8.5)	24.6 (11.2)
Number of past quit attempts, mean (SD)	3.5 (3.2)	3.5 (3.1)
Number of cohabitant smokers, mean (median)	1.1 (1)	0.5 (0)
Smoking duration (years), mean (SD)	17.3 (5.0)	27.0 (10.8)
Past or current nicotine replacement therapy usage, n (%)	50 (52%)	33 (26%)

### Comparison of SHO and SSC Superusers

A multivariate logistic regression revealed no statistically significant differences in demographic characteristics between the two populations. Gender, age, cigarettes per day, past quit attempts, cohabitant smokers, years smoked, and past or current use of nicotine replacement therapy were all nonsignificant.

### Superuser Subgroup Analysis

To gain further understanding of superuser and superuser-subset characteristics, we determined Pearson correlations to investigate associations between posting behavior and five key demographic characteristics (age, cigarettes smoked per day, quit attempts, cohabitant smokers, and years smoked).

#### SHO Pearson Correlations

In the SHO analysis (see Table 5), a statistically significant correlation was revealed between posting behavior and age for all superusers. However, the Pearson correlation was .32, indicating only a small correlation. The only other significant correlation was among clique members, where there was a statistically significant correlation between posting behavior and years smoked. However, this instance also indicated only a small correlation.

**Table 5:** Bivariate correlations between Smokers’ Helpline Online superuser type and demographic characteristics.

Variable	All superusers *r *(*P *value)	Posters *r* (*P *value)	Thread starters *r* (*P *value)	Clique members *r* (*P *value)
Age	.32 (.002)	.22 (.08)	.17 (.18)	.23 (.07)
Cigarettes per day	.05 (.66)	.06 (.66)	–.02 (.90)	.06 (.66)
Quit attempts	–.07 (.52)	–.07 (.58)	.03 (.83)	–.06 (.62)
Cohabitant smokers	.03 (.74)	.16 (.21)	.18 (.16)	.20 (.12)
Years smoked	.09 (.37)	.13 (.27)	.14 (.17)	.27 (.03)

#### SSC Pearson Correlations

In the SSC analysis (see Table 6), there were statistically significant correlations between posting behavior, quit attempts, and years smoked among all superusers, posters, and clique members. However, these relationships were relatively small (*r *ranging from –.25 to .25). There were no correlations between posting behavior and demographic characteristics among thread starters.

**Table 6:** Bivariate correlations between StopSmokingCenter.net superuser type and demographic characteristics.

Variable	All superusers *r* (*P *value)	Posters *r* (*P *value)	Thread starters *r* (*P *value)	Clique members *r* (*P *value)
Age	.03 (.73)	.07 (.46)	.03 (.76)	.07 (.52)
Cigarettes per day	.12 (.18)	.13 (.21)	.07 (.52)	.18 (.08)
Quit attempts	–.25 (.01)	–.24 (.02)	–.16 (.11)	–.23 (.02)
Cohabitant smokers	–.08 (.40)	–.02 (.90)	.02 (.82)	–.04 (.71)
Years smoked	.24 (.01)	.25 (.01)	.18 (.07)	.25 (.01)

## Discussion

At first glance, based on general demographic characteristics outlined in Table 2, SSC superusers appear to have smoked much longer than SHO superusers, and a smaller number had used or were using nicotine replacement therapy. From these data, marketers and social network health educators may infer that SHO superusers started smoking later in life, and over half have used or are using nicotine replacement therapy.

However, contrary to our original hypothesis, and to information gleaned from informal qualitative in-person interviews with health educators, differences in marketing and the operation of both social networks, tone of discussions, and rules of conduct, superusers in both social networks had similar demographic characteristics.

We were also somewhat surprised to find minimal correlations between posting behavior and demographic characteristics in all eight of the superuser groups analyzed. For example, a Health Canada general population survey found correlations in demographic characteristics and behavior. Although there was little difference in quit rates between Canadian men and women, 29% of smokers aged 20–24 years had quit versus 71% of those aged 45+ years, former male smokers reported an average of 3.2 quit attempts before quitting for good (versus 2.7 quit attempts for females), and at the time of quitting, former smokers reported smoking 18.1 cigarettes per day [38].

Based on the Health Canada general population survey, one might expect to find strong correlations between frequency of posts and age, past quit attempts, or number of cigarettes smoked per day among superusers. Instead, we found only weak relationships between posting behavior, quit attempts, and years smoked, but only in SSC superusers, posters, and clique members.

### Strengths and Limitations

To our knowledge, this is the first study that compared demographic characteristics and posting behavior of the most active participants (superusers) from two moderated social networks designed to assist with smoking cessation. A particular strength is that both programs have relatively few barriers to enrollment in comparison with entry barriers that are typical of Internet-based clinical trials [39], as we collected data from WATI operating in their naturalistic Internet environments.

Another strength is the applicability of findings. Results from this study will influence SHO promotional efforts and operations. Web promotion is generally directed at specific and targeted demographics, such as those reported in the Health Canada general population survey. However, to attract superusers, who are not easily defined, promotional efforts should be broadened. Second, as a results of this study management will modify the training programs of SHO and SSC health educators. Health educators will be introduced to strategies that encourage the participation of superusers once identified within the network, and work is underway to develop computer algorithms that will assist health educators with the early identification of superusers and superuser subtypes.

It is important to note that we have used the term superuser and the three superuser subgroups (posters, thread starters, and clique members) only for the purpose of clarifying observations within this specific investigation. These participatory patterns have not been validated, and further research is required to determine whether they are observable across other types of WATI or health care-based social networks.

To more thoroughly understand superuser characteristics, future studies should compare superuser populations versus nonsuperuser populations (or social network members who create limited posts) and those who register with WATI but do not actively participate in the social network (lurkers). Future research studies should also incorporate the analysis of additional demographic and psychographic characteristics such as occupation, level of education, Goldberg Depression Scale score, Fagerström Test for Nicotine Dependence score, or frequency of Internet usage. These data may be collected at registration; however, in our experience, questions presented to users at registration are most often regarded as intrusive. To maximize use of Web-based programs in a naturalistic setting, registration questions should be kept to an absolute minimum, and other creative means of data collection should be used within interactive program content.

It is also important to note that this study focused only on smokers, and future studies should examine social network behavior and demographic characteristics from superusers, superuser subsets, moderate posters, and lurkers from other condition areas such as depression, panic disorder, problem drinking, self-harm, or healthy weight. This type of research may offer insight into general user characteristics for those who are attracted to Internet programs, and may be used in the development of predictive algorithms.

### Summary

The results of this study indicate that superusers play powerful roles within social network traffic. While SHO and SSC superusers accounted for only 0.4% and 1.1% of total program registrants, they were responsible for 34.78% and 46.22% of social network content. The results of this study support the previous research of Cobb et al, Selby et al, Cunningham et al, and Jones et al, indicating that different types of superusers (and superuser subgroups) frequently exist.

Significant and well-recognized barriers to treatment include social stigmas that prevent treatment seeking [40] and inconveniences involved with physically attending treatment or in-person group therapy [41]. Whether individuals act as superusers or passively read the posts from others, the Internet’s ability to anonymously and conveniently reach large numbers of individuals can significantly affect health on a population level.

The results of this study suggest that further research in this fast-growing field is required, and that there is potential to maximize the impact of social networks that promote wellness. However, to fully understand the unique mechanisms of Internet-based behavior change, collaboration and knowledge transfer between researchers, nonprofit organizations, and private organizations is recommended.

## Abbreviations

SHO: Smokers’ Helpline Online
SSC: StopSmokingCenter.net
WATI: Web-assisted tobacco interventions
